# The Biofilm Inhibitor Carolacton Enters Gram-Negative Cells: Studies Using a TolC-Deficient Strain of Escherichia coli

**DOI:** 10.1128/mSphereDirect.00375-17

**Published:** 2017-09-27

**Authors:** Jannik Donner, Michael Reck, Boyke Bunk, Michael Jarek, Constantin Benjamin App, Jan P. Meier-Kolthoff, Jörg Overmann, Rolf Müller, Andreas Kirschning, Irene Wagner-Döbler

**Affiliations:** a Department of Medical Microbiology, Group Microbial Communication, Helmholtz-Centre for Infection Research, Braunschweig, Germany; b Leibniz Institute DSMZ-German Collection of Microorganisms and Cell Cultures, Braunschweig, Germany; c German Centre for Infection Research (DZIF), Partner Site Hannover–Braunschweig, Braunschweig, Germany; d Genome Analytics, Helmholtz Centre for Infection Research, Braunschweig, Germany; e Department of Microbial Natural Products, Helmholtz Institute for Pharmaceutical Research Saarland (HIPS), Helmholtz Centre for Infection Research and Pharmaceutical Biotechnology, Saarland University, Saarbrücken, Germany; f Institute of Organic Chemistry and Center of Biomolecular Drug Research (BMWZ), Leibniz Universität Hannover, Hannover, Germany; Antimicrobial Development Specialists, LLC; University Medicine Rostock; University of Kansas Medical Center; Arizona State University

**Keywords:** Gram-negative bacteria, antimicrobial activity, antimicrobial agents, carolacton, drug efflux, drug resistance mechanisms, efflux pumps, gene sequencing, genome analysis

## Abstract

The myxobacterial secondary metabolite carolacton inhibits growth of Streptococcus pneumoniae and kills biofilm cells of the caries- and endocarditis-associated pathogen Streptococcus mutans at nanomolar concentrations. Here, we studied the response to carolacton of an Escherichia coli strain that lacked the outer membrane protein TolC. Whole-genome sequencing of the laboratory E. coli strain TolC revealed the integration of an insertion element, IS*5*, at the *tolC* locus and a close phylogenetic relationship to the ancient E. coli K-12. We demonstrated via transcriptome sequencing (RNA-seq) and determination of MIC values that carolacton penetrates the phospholipid bilayer of the Gram-negative cell envelope and inhibits growth of E. coli TolC at similar concentrations as for streptococci. This inhibition is completely lost for a C-9 (*R*) epimer of carolacton, a derivative with an inverted stereocenter at carbon atom 9 [(*S*) → (*R*)] as the sole difference from the native molecule, which is also inactive in S. pneumoniae and S. mutans, suggesting a specific interaction of native carolacton with a conserved cellular target present in bacterial phyla as distantly related as *Firmicutes* and *Proteobacteria*. The efflux pump inhibitor (EPI) phenylalanine arginine β-naphthylamide (PAβN), which specifically inhibits AcrAB-TolC, renders E. coli susceptible to carolacton. Our data indicate that carolacton has potential for use in antimicrobial chemotherapy against Gram-negative bacteria, as a single drug or in combination with EPIs. Strain E. coli TolC has been deposited at the DSMZ; together with the associated RNA-seq data and MIC values, it can be used as a reference during future screenings for novel bioactive compounds.

**IMPORTANCE** The emergence of pathogens resistant against most or all of the antibiotics currently used in human therapy is a global threat, and therefore the search for antimicrobials with novel targets and modes of action is of utmost importance. The myxobacterial secondary metabolite carolacton had previously been shown to inhibit biofilm formation and growth of streptococci. Here, we investigated if carolacton could act against Gram-negative bacteria, which are difficult targets because of their double-layered cytoplasmic envelope. We found that the model organism Escherichia coli is susceptible to carolacton, similar to the Gram-positive Streptococcus pneumoniae, if its multidrug efflux system AcrAB-TolC is either inactivated genetically, by disruption of the *tolC* gene, or physiologically by coadministering an efflux pump inhibitor. A carolacton epimer that has a different steric configuration at carbon atom 9 is completely inactive, suggesting that carolacton may interact with the same molecular target in both Gram-positive and Gram-negative bacteria.

## Abstract (precis)

The emergence of pathogens resistant against most or all of the antibiotics currently used in human therapy is a global threat, and therefore the search for antimicrobials with novel targets and modes of action is of utmost importance. The myxobacterial secondary metabolite carolacton had previously been shown to inhibit biofilm formation and growth of streptococci. Here, we investigated if carolacton could act against Gram-negative bacteria, which are difficult targets because of their double-layered cytoplasmic envelope. We found that the model organism Escherichia coli is susceptible to carolacton, similar to the Gram-positive Streptococcus pneumoniae, if its multidrug efflux system AcrAB-TolC is either inactivated genetically, by disruption of the *tolC* gene, or physiologically by coadministering an efflux pump inhibitor. A carolacton epimer that has a different steric configuration at carbon atom 9 is completely inactive, suggesting that carolacton may interact with the same molecular target in both Gram-positive and Gram-negative bacteria.

## INTRODUCTION

The identification of novel compounds for antimicrobial chemotherapy is becoming increasingly difficult (1). This is especially true for compounds targeting Gram-negative bacteria, for two main reasons: first, the second outer plasma membrane of Gram-negative organisms acts as a potent barrier and restricts the entry of hydrophilic extracellular substances, such as antibiotics, into the cell (2); second, the multidrug resistance (MDR) efflux systems present in many Gram-negative bacteria provide intrinsic resistance against antibiotics (3). The primary function of MDR efflux systems is the removal of toxins and bile acids from the cytoplasm, which is important for infectivity and virulence (4). MDR facilitated by extrusion of antibiotics has become a serious problem in the treatment of infections by, e.g., Escherichia coli (5), Klebsiella pneumoniae (6), Pseudomonas aeruginosa (7), and Salmonella enterica (8).

Proton-dependent tripartite envelope translocase systems (TETS) are widely distributed MDR efflux systems which have been studied extensively in E. coli and P. aeruginosa. TETS characteristically consist of an MDR pump, a membrane fusion protein (MFP), and an outer membrane factor (OMF) (9). In E. coli, MDR pumps of the resistance-nodulation-division (RND) family are key contributors to intrinsic antibiotic resistance (10). The genome of E. coli includes six genes for MDR pumps of the RND family (*acrB*, *acrF*, *yhiU*, *acrD*, *yegN*, and *yegO*) and seven genes for MFPs (*acrA*, *acrE*, *yhiV*, *yegM*, *emrA*, *emrK*, and *ybjY*) (11). As the third component of tripartite efflux systems, E. coli possesses four genes encoding OMF proteins, *tolC*, *mdtP*, *mdtQ*, and *cusC*, which are essential for a functional RND pump (e.g., AcrA-AcrB-TolC) (11). Among all OMF proteins of E. coli, TolC appears to be the major facilitator for extrusion of antibiotics and small molecules through the outer membrane (11, 12). In particular, the AcrAB-TolC tripartite efflux system is of great scientific interest, since it is constitutively expressed, has a broad substrate specificity, is found in a wide variety of clinically relevant Gram-negative pathogens (e.g., P. aeruginosa, S. enterica, and *Klebsiella* spp.), and contributes to MDR (4).

Therefore, efflux pump inhibitors (EPIs) can be important for the discovery of novel antibiotics (13), and they can be applied in combination with current antibiotics to overcome extrusion by MDR efflux systems (13, 14). Among them, the peptidomimetic EPI phenylalanine arginine β-naphthylamide (PAβN; MC-2077110) (15) was found to specifically block the AcrAB- and AcrEF-based MDR efflux systems in E. coli, which are both dependent on TolC as the OMF (16). On the other hand, bacterial strains with defects in MDR efflux systems are often used as sensitive indicators for antimicrobial activity (17). TolC mutants of E. coli, for example, are hypersensitive to 19 of 22 antibiotics tested (12).

The screening of libraries of natural secondary metabolites holds great promise for the discovery of novel antimicrobial compounds (18). During such screenings, the myxobacterial macrolide ketocarbonic acid carolacton was identified as a biofilm inhibitor (19, 20). Its activity against clinically relevant streptococci was later analyzed in great detail (20–24). The exact molecular target of carolacton remains unknown, but the complete loss of biological activity of a carolacton epimer at C-9 [(*S*) → (*R*)] (*epi*-carolacton) in Streptococcus mutans biofilms and planktonically growing Streptococcus pneumoniae cells (22, 24) suggests an interaction of carolacton with a conserved cellular target (24).

Carolacton is inactive against E. coli (MIC, >40 µg/ml), but strong growth inhibition was found when a laboratory E. coli strain recorded as lacking a functional copy of the OMF TolC (E. coli TolC) was treated with carolacton (MIC, 0.06 µg/ml) (19). These data suggested that carolacton might be able to pass through the Gram-negative cell envelope and that the lack of sensitivity of wild-type E. coli to carolacton is due to export from the cell by TolC-mediated efflux. However, mutations in TolC can have different effects on substrate export, and there have even been reports that a misassembled TolC protein may result in an open channel which allows influx of antibiotics into the cell, resulting in an increased sensitivity (25). The TolC-deficient strain used in our screenings has been propagated as a glycerol stock in laboratories since at least 1980 (B. Kunze, personal communication), and so far it has not been characterized genetically. Over a period of 37 years, massive genetic changes could have occurred (26). Moreover, although TolC-deficient strains are used by many laboratories, they were constructed with different methods and in different genetic backgrounds (25, 27, 28), making it hard to compare results. We here determined the genome sequence of E. coli TolC with high resolution by using a combination of PacBio and Illumina sequencing. With these methods, an insertion of a natural transposon at the *tolC* locus was identified, and genetic changes were recorded that had occurred in this strain in comparison to its closest relative, which was identified as E. coli K-12 MG1655 (NZ_CP014225.1). We determined MICs for E. coli K-12 MG1655 and E. coli TolC and deposited E. coli TolC with the DSMZ as a tool and reference for future studies. We then studied the influence of carolacton on E. coli TolC by using transcriptome sequencing (RNA-seq), the carolacton C-9 (*R*) epimer, and the EPI PAβN. The data clearly showed that carolacton easily penetrates the Gram-negative cell envelope. Once inside the cell, it inhibits E. coli at similar concentrations as for streptococci, suggesting that the molecular target of carolacton is highly conserved and might be highly similar even in distantly related bacterial phyla, such as *Firmicutes* and *Proteobacteria*. The export of carolacton from the cell can be overcome by blocking the AcrAB-TolC efflux complex with the EPI PAβN. This finding highlights the potential use of carolacton in combinatorial treatment with EPIs.

## RESULTS

### E. coli TolC is an ancient natural derivative of E. coli K-12 and is closely related to K-12 MG1655.

PacBio single-molecule real-time (SMRT) sequencing and Illumina MiSeq short-read sequencing were combined to obtain a high-quality genome sequence of E. coli TolC. By Illumina MiSeq sequencing, 2,623,454 reads were obtained, totaling ~656 Mb and resulting in ~138-fold genome coverage. The PacBio SMRT sequencing data set consisted of 74,571 reads with an N50 read length of 17,770 bp and was used for *de novo* genome assembly. For the correction of indel errors, Illumina reads were mapped onto the newly assembled genome.

The genome of E. coli TolC (CP018801.1) consists of a single chromosome that is 4,792,200 bp long and contains 4,469 coding sequences (CDS), 88 tRNAs, 22 rRNAs, and 104 noncoding RNAs (ncRNAs). It was compared to all 259 fully sequenced E. coli genomes available from the National Center for Biotechnology Information (NCBI) via *in silico* DNA-DNA hybridization (*is*DDH), with *is*DDH values calculated by using the tool GGDC 2.1 (29). E. coli TolC showed the highest *is*DDH values (all *is*DDH values ≥98.28%) to E. coli strains K-12 MG1655 (NZ_CP014225.1), ER1821R (NZ_CP016018.1), NCM3722 (NZ_CP011495.1), K-12 W3110 (NC_007779.1), and JW5437-1 (NZ_CP014348.1).

A nucleotide-based genome BLAST distance phylogeny (GBDP) tree with branch support values inferred from both the nucleotide and amino acid data is depicted in Fig. S1 of our supplementary data posted on figshare (https://doi.org/10.6084/m9.figshare.5395471). The average branch support of the nucleotide tree was 47.3%, and branch support for the amino acid tree was 37.6%. Target strain E. coli TolC was placed in a highly supported subtree containing 14 strains, most of them K-12 strains.

**FIG 1:**
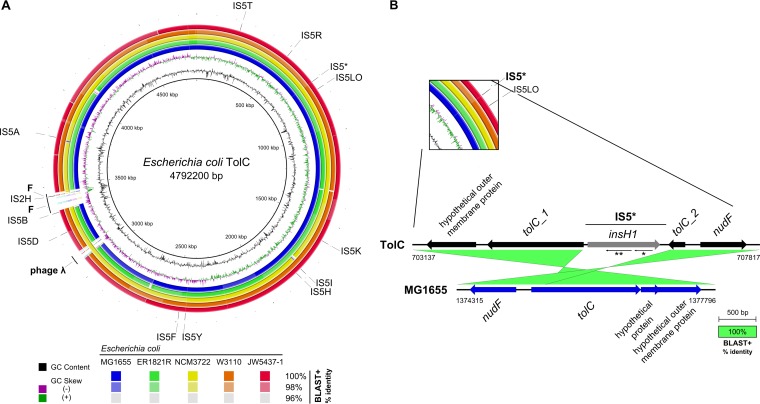
Whole-genome comparison of E. coli TolC to closely related strains and a schematic presentation of transposon-mediated disruption of the *tolC* CDS in E. coli TolC. (A) BLAST ring image generator (BRIG) (64) comparison of the E. coli TolC genome (innermost black ring) to the closely related genomes of E. coli strains K-12 MG1655, ER1821R, NCM3722, K-12 W3110, and JW5437-1 (the four outermost rings), shown in blue to red, respectively, as identified by *is*DDH (29). Shading of the four outermost rings is according to their respective percent nucleotide identity to the query sequence (E. coli TolC), determined by BLAST+. The second and third innermost rings show the GC skew (purple/green) and the GC content (black). IS*5* elements are numbered according to annotations for E. coli K-12 MG1655 (NC_000913.3). The location of the fertility plasmid on the chromosome of E. coli TolC is indicated by the letter F (on left side of diagram). (B) Close-up comparison of the *tolC* locus of E. coli TolC and its closest relative, E. coli K-12 MG1655, drawn by using Easyfig (66) The *tolC* locus (*tolC_1* and *tolC_2*) in E. coli TolC is interrupted by insertion of an IS*5* element (IS5*) that codes for the transposase *insH1* (*ins5A*). *ins5B* (**) and *ins5C* (*) are indicated by arrows in reverse orientation, underneath *insH1*. A BLAST+ comparison of the *tolC* locus for each of the two strains indicated 100% nucleotide identity.

**FIG 2:**
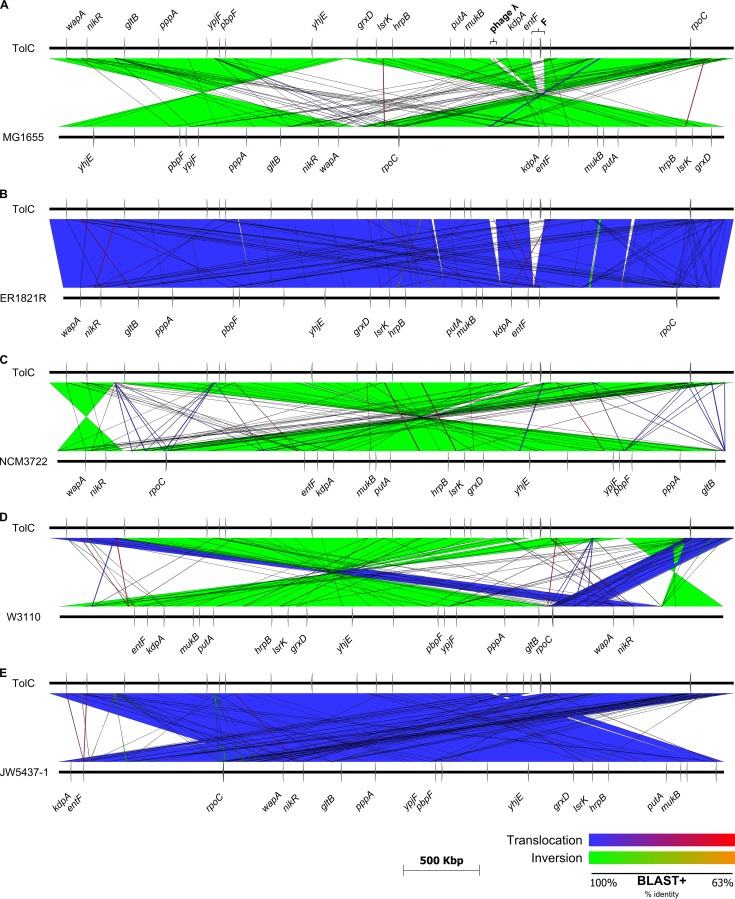
Genomic rearrangements of E. coli TolC in comparison with the most closely related strains. The complete genome of E. coli TolC was compared to the genomic sequences of E. coli K-12 MG1655 (A), E. coli ER1821R (B), E. coli NCM3722 (C), E. coli K-12 W3112 (D), and E. coli MG1655 JW5437-1 (E), and structural rearrangements were visualized using Easyfig (65). The relative locations of individual reference genes (in comparison to E. coli *tolC* in panel A) are indicated by gray arrows on the respective chromosomes (black horizontal lines). The nucleotide sequence identities, as determined using BLAST+, are indicated by different colored spectra: blue to red for translocations, and green to orange for inversions. Blue/green and red/orange indicate the highest (100%) and lowest (63%) detected sequence identities, respectively.

Figure 1 shows the nucleotide sequence identity of E. coli TolC in comparison to the five most similar E. coli strains, as reported in BLAST+. Most notably, E. coli TolC contains the bacteriophage λ and the fertility plasmid F integrated into its chromosome. Phage λ was located between genes *ybhB* and *ybhC* at positions 3,079,545 to 3,128,200 of the E. coli TolC chromosome, and the F plasmid was integrated into an insertion sequence element (IS*3C*) within the cryptic prophage DLP12 (positions 3,368,702 to 3,467,447). This is in contrast to the most closely related E. coli strains, which encode neither the fertility plasmid nor phage λ, the only exception being NCM3722, which still carries phage λ (Fig. 1A). In comparison to MG1655, an* rph-1* mutation is absent in TolC, and the *rpoS* gene is present as the 33Am variant. Like other derivatives of E. coli K-12, strain E. coli TolC is also valine sensitive (*ilvG* deficient) (30). Similar to E. coli MG1655, an early deletion of two nucleotides (c.977_978delAT) that results in an Ile327-Glu substitution and subsequent insertion of a premature TGA translation termination site at position c.982_984 were found. As a common marker of all E. coli K-12 derivatives, E. coli TolC additionally carries an IS*5* insertion (IS*5*I) in the last gene of the O-antigen cluster encoding the rhamnosyltransferase WbbL (*rfb-50* mutation) (31). Although these strains are closely related, large structural rearrangements within their chromosomes were found (Fig. 2).

The *tolC* locus (*btd92_00696*) was inspected in detail, and the absence of a functional copy of the *tolC* gene was confirmed. The E. coli TolC strain carries a transposon insertion after base 1309 (c.1309_1310insIS5*) of the *tolC* gene, and this causes a disruption of the CDS (Fig. 1B). Genes of the three additional OMF proteins in E. coli (*cusC*, *mdtQ*, and *mdtP*) were not affected (see Table S1 at https://doi.org/10.6084/m9.figshare.5395471). The transposon within *tolC* was identified as transposable element IS*5*, which contains three protein-coding genes: the transposase gene *insH1* (*ins5A*) and two genes (*ins5B* and *ins5C*) opposite *insH1* with unknown function (32, 33). Altogether, the E. coli TolC chromosome contained 12 insertions of IS*5* elements, of which only the one integrated into the *tolC* locus (IS5*) disrupted a functional gene. Additionally, IS*5* insertions were also located within the sequences of cryptic prophages, e.g., the IS*5*Y element was inserted into the cryptic prophage Rac, interrupting *lomR*′. The E. coli TolC strain described here was deposited at the Leibniz Institute DSMZ German Collection of Microorganisms and Cell Cultures (Braunschweig, Germany) and assigned strain number DSM 104619.

### Role of TolC for MICs of carolacton and different classes of antibiotics.

To evaluate the effect of TolC inactivation on antibiotic susceptibility of E. coli, the MICs of selected antibiotics against E. coli MG1655 and E. coli TolC were determined (Table 1). We included two RNA polymerase inhibitors, corallopyronin A and sorangicin, previously isolated from myxobacteria at our institution (34, 35).

**TABLE 1:** MICs of antibiotics and carolacton against E. coli TolC and E. coli K-12 MG1655

Mechanism and/or antibiotic	Target	MIC (µg/ml)^a^		FC^b^
		E. coli K-12 MG1655	E. coli TolC	
Carolacton		>8	**0.125**	64
Carolacton with (40 µg/ml PAβN)		4	**≤0.03**	128
Protein biosynthesis				
Chloramphenicol	50S ribosomal subunit	8	**1**	8
Erythromycin	50S ribosomal subunit	>64	**2**	32
Gentamicin	30S ribosomal subunit	4	2	2
Kanamycin	30S ribosomal subunit	8	4	2
Peptidoglycan biosynthesis				
Ampicillin	Penicillin-binding proteins	16	**4**	4
Cephalotin	Penicillin-binding proteins	16	8	2
Cefotaxime	Penicillin-binding proteins	0.0625	**0.015**	4
Penicillin G	Penicillin-binding proteins	>32	16	2
Vancomycin	d-Ala-d-Ala moieties of NAM/NAG^c^ peptides	>256	>256	1
Phosphomycin	UDP-*N*-acetylglucosamine-3-enolpyruvyltransferase (MurA)	>32	**4**	8
Fatty acid biosynthesis				
Triclosan	Enoyl-acyl carrier protein reductase (FabI)	0.125	**≤0.0078**	16
Cerulenin	β-keto-acyl-ACP synthase (FabB)	>32	**4**	8
RNA biosynthesis				
Corallopyronin A	RNA polymerase	>32	**2**	16
Rifampin	RNA polymerase	16	8	2
Sorangicin	RNA polymerase	16	16	1
Cell division				
Novobiocin	DNA gyrase	>16	**1**	16
Ciprofloxacin	DNA gyrase	0.015	**0.0039**	4
Folate biosynthesis				
Trimethoprim	Dihydrofolate reductase (FolA)	0.5	**0.063**	8
Sulfamethoxazole	Dihydropteroate synthase (FolP)	128	64	2

a Boldface values indicate that MICs for the MG1655 control strain differed by ≥4-fold.

b The FC increase in susceptibility of E. coli TolC relative to E. coli MG1655 susceptibility.

c NAM, *N*-acetylmuramic acid; NAG, *N*-acetylglucosamine.

E. coli TolC was at least 64 times more sensitive to carolacton than E. coli MG1655. The MIC of carolacton against E. coli TolC was in the same range as that reported by Jansen et al. (19). For S. pneumoniae TIGR4, the MIC of carolacton was determined to be 0.06 µg/ml (24), similar to the value reported for E. coli TolC. In comparison to E. coli MG1655, E. coli TolC showed a strong increase in sensitivity (≥4-fold) to antibiotics from all functional groups. The determined MICs were in the same range as those reported previously for E. coli W3110 and its *tolC* null mutant (11), indicating that the presence of the F plasmid and phage λ do not affect antibiotic susceptibility. Rifampin and vancomycin are not substrates of the pump; thus, E. coli TolC is not expected to be hypersensitive to these compounds, which was confirmed. The data indicated that carolacton penetrates the two membranes of the Gram-negative cell envelope and that its intracellular inhibitory effect is comparable to that of Gram-positive cells.

### Transcriptional response of E. coli TolC to carolacton.

We analyzed the transcriptome of carolacton-treated cultures of E. coli TolC in comparison to untreated cultures during the first 30 min of growth.

**FIG 3:**
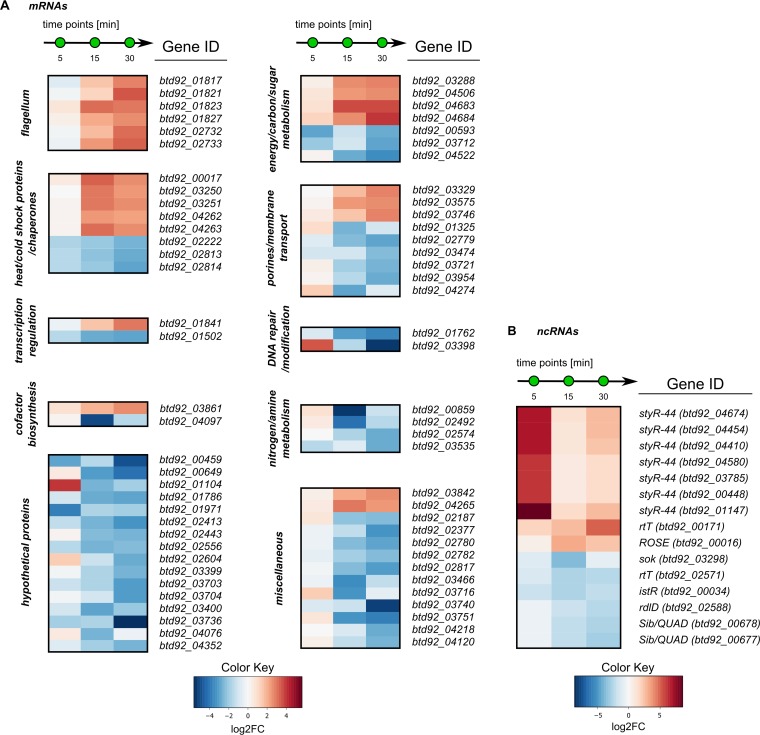
The most strongly differentially abundant transcripts in E. coli TolC during growth with carolacton (0.25 µg/ml). (A) Overview; (B) the most strongly differently regulated ncRNAs. The cutoff for differentially abundant transcripts was set at log_2_ FC of ≥ ±2 for general transcripts and ≥ ±2.5 for ncRNAs (FDR, ≤0.01), for at least one sample during the time course.

In total, 4,730 transcripts of E. coli TolC were investigated using Rockhopper (see Data Set S1 in the supplemental material). At 30 min after addition of carolacton, 71 transcripts showed a strong differential abundance (log_2_ fold change [FC] of ≥±2), corresponding to 1.6% of all open reading frames of E. coli TolC (Data Set S2). At this time point, E. coli TolC grows at the same rate with or without carolacton (see below). The data therefore provide additional proof that carolacton immediately enters the Gram-negative cell. At a log_2_ FC of ≥±0.8, approximately 29% of all genes were differentially abundant, comparable to the degree of differential transcript abundance in S. mutans (31.3%) and S. pneumoniae (22.8%) in the presence of carolacton when we used an identical cutoff (21, 24). The most strongly differentially abundant transcripts encoded components for flagellar assembly, heat shock and cold shock proteins, and chaperones (Fig. 3). Transcription of the alternative sigma factor F (σ^28^) was upregulated ~7.4-fold (log_2_ FC, 2.88), and the putative helix-turn-helix (HTH)-type transcriptional regulator RhmR was downregulated. Moreover, precursors of the outer membrane pore proteins NmpC (*btd92_03329*) and PhoE (*btd92_03746*) were upregulated. Interestingly, all 7 StyR-44 family small noncoding RNAs encoded in the genome were strongly (log_2_ FC, ≥6.5) upregulated after only 5 min of growth with carolacton. The data showed that interaction of E. coli TolC with carolacton triggers global transcriptional adaptations already after 5 min, suggesting a molecular target in a central metabolic pathway.

10.1128/mSphereDirect.00375-17.1 DATA SET S1 Log_2_ FC and FDR values for transcripts of E. coli TolC (CP018801). Log_2_ FC and FDR values were obtained from analyzing differential transcription with the edgeR package (v.3.1) for R. Download DATA SET S1, XLSX file, 0.5 MB. Copyright © 2017 Donner et al. 2017 Donner et al. This content is distributed under the terms of the Creative Commons Attribution 4.0 International license.

10.1128/mSphereDirect.00375-17.2 DATA SET S2 (A) Log_2_ FC and FDR values of the most differentially regulated (log_2_ FC ≥ ±2.5) protein-coding transcripts of E. coli TolC (CP018801) when grown with carolacton, calculated by using the edgeR package (v.3.1) for R. (B) Log_2_ FC and FDR values of the most differentially regulated (log_2_ FC ≥ ±2.5) ncRNAs of E. coli TolC (CP018801) when grown with carolacton, calculated by using the edgeR package (v.3.1) for R. Download DATA SET S2, XLSX file, 0.02 MB. Copyright © 2017 Donner et al. 2017 Donner et al. This content is distributed under the terms of the Creative Commons Attribution 4.0 International license.

### Stereospecificity of carolacton activity and inhibition of efflux.

**FIG 4:**
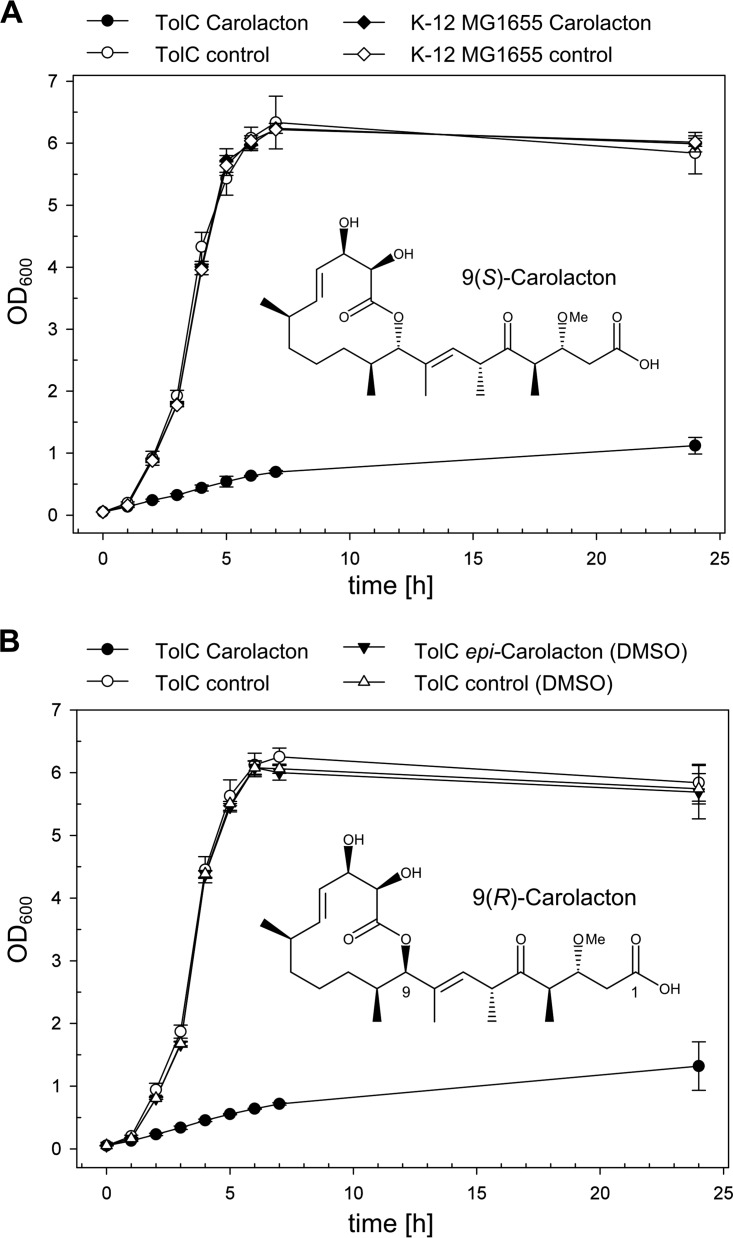
Growth inhibition of E. coli TolC and E. coli K-12 MG1655 by carolacton and sensitivity of E. coli TolC to *epi*-carolacton. (A) Chemical structure of native 9(*S*) carolacton and growth inhibition of E. coli TolC and E. coli K-12 MG1655 with 9(*S*) carolacton. (B) Structure of 9(*R*) carolacton (*epi*-carolacton) and inhibition activity against E. coli TolC treated with carolacton-methanol (circles) or in the presence of *epi*-carolacton and DMSO (triangles) for 24 h. Growth curves represent the mean (and standard deviation) results of three independent experiments, Carolacton was added at a final concentration of 0.25 µg/ml at *t* = 0 min.

**FIG 5:**
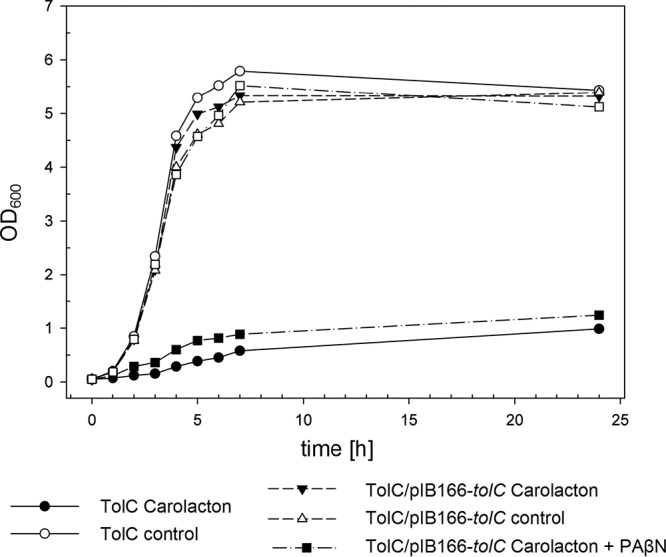
Complementation of the E. coli TolC strain with a plasmid-carried copy of *tolC*. For expression of a functional copy of TolC in E. coli TolC, the *tolC* gene and native regulatory sequences were PCR amplified from E. coli MG1655 and cloned into pIB166, and the resulting construct was transformed into E. coli TolC. E. coli TolC/pIB166-*tolC* was grown with 20 µg/ml chloramphenicol, and the AcrAB-TolC efflux pump inhibitor PAβN was applied at a final concentration of 40 µg/ml. The growth curves are representative of results for three biological replicates.

Subsequently, the differences in carolacton susceptibility between E. coli TolC and E. coli MG1655 were investigated in detail over all growth phases. E. coli MG1655 with and without carolacton and TolC without carolacton grew similarly and reached their maximal optical density at 600 nm (OD_600_) of ~6 after 7 h (Fig. 4A). In the presence of carolacton (added at *t* = 0), growth was indistinguishable from the controls for 1 h. At this time point, growth of the carolacton-treated culture of the E. coli TolC strain was strongly inhibited, while all other strains entered the exponential growth phase. The carolacton-treated culture of the E. coli TolC strain grew linearly over the next 5 h to an OD_600_ of approximately 0.8, which did not increase much farther and reached a maximal OD_600_ of around 1 after 24 h. Complementation of E. coli TolC with a plasmid-borne copy of the OMF TolC was able to restore insensitivity to carolacton, confirming indeed the absence of TolC-mediated efflux of carolacton as the sole cause for sensitivity (Fig. 5).

*epi*-Carolacton is a carolacton epimer with an inversion of the stereocenter at C-9 from the native (*S*) to the (*R*) configuration. This carolacton derivative lacks biological activity in S. pneumoniae TIGR4 and S. mutans UA159 (22, 24). Here, we tested the inhibitory properties of *epi*-carolacton against E. coli TolC. Figure 4B shows that *epi*-carolacton had no influence on growth of E. coli TolC. Since *epi*-carolacton was dissolved in dimethyl sulfoxide (DMSO), we investigated its effect on growth as an additional control, but we did not detect any. The loss of growth inhibition of *epi*-carolacton shown here suggests that the molecular target of carolacton might not only be conserved in the genus *Streptococcus* but also in the phyla *Firmicutes* and *Proteobacteria*.

Antibiotics that are substrates of TolC have to be administered in high doses to overcome the intrinsic resistance mediated by efflux (13). Alternatively, they could be applied in combination with efflux pump inhibitors. Therefore, we investigated the influence of PAβN, a competitive inhibitor of AcrAB-TolC (16), on carolacton sensitivity in E. coli. Table 1 shows that the MIC of E. coli MG1655 toward carolacton was reduced from >8 µg/ml to 4 µg/ml when PAβN was coadministered at 40 µg/ml. Lower concentrations of PAβN had no effect on the MIC of carolacton. The susceptibility of the TolC mutant was also increased by PAβN. The MIC of E. coli against PAβN has been shown before to be strongly reduced in an efflux-deficient strain (Δ*acrAB*); moreover, PAβN can cause membrane destabilization as an unspecific side effect (16). Accordingly, we observed a growth reduction of ~45% for the efflux-deficient E. coli TolC strain when grown with 40 µg/ml PAβN, but not for the wild-type (Fig. 5).

**FIG 6:**
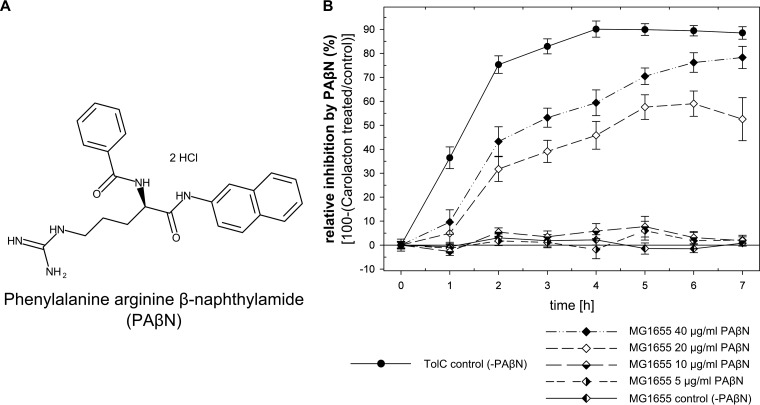
Inhibition of the AcrAB-TolC complex by PAβN leads to susceptibility of wild-type E. coli to carolacton. (A) Structure of the EPI PAβN. (B) Relative growth inhibition of E. coli TolC and E. coli K-12 MG1655 by carolacton in the presence of PAβN, as the percentage of control growth. The relative inhibition was calculated by dividing the OD of the carolacton-treated culture by the OD of the untreated control for every time point; both cultures contained the indicated amount of PAβN. Data show means and standard deviations for results from three biological replicates.

Finally, we investigated the role of PAβN (Fig. 6A) under the same conditions as those used for studying the effect of TolC deletion. The effect of PAβN on growth inhibition of E. coli MG1655 by carolacton was dependent on the concentration of PAβN used (Fig. 6B). At concentrations of 20 and 40 µg/ml PAβN, a maximal inhibition of 59% and 78%, respectively, was found, in comparison to a culture treated with only carolacton. The observations concerning MICs and a PAβN-mediated growth inhibition by carolacton were reproducible for the *tolC*-complemented E. coli TolC strain (Table 2 and Fig. 5, respectively). For comparison, inhibition of growth of E. coli TolC treated with carolacton is shown, which reached a maximum of 90% in comparison to the untreated culture (Fig. 6B). Thus, in E. coli, addition of 40 µg/ml PAβN, together with carolacton, causes a growth reduction similar to that with treatment with carolacton in a TolC-deficient strain.

**TABLE 2:** MICs of carolacton against a *tolC*-complemented strain of E. coli TolC

Strain	MIC of carolacton (µg/ml)
E. coli TolC/pIB166-*tolC*	>8
E. coli TolC/pIB166-*tolC* (with 40 µg/ml PAβN)	2

**FIG 7:**
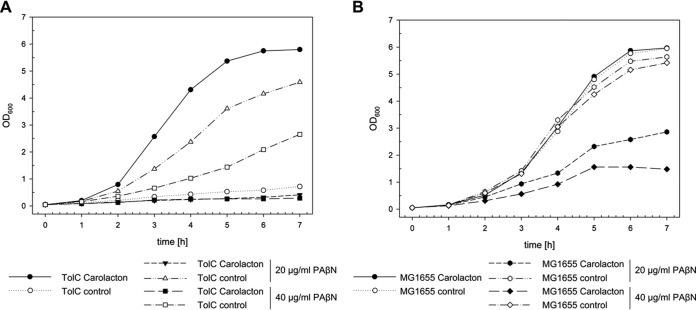
Effect of carolacton in combination with PAβN on growth of E. coli strains. (A) Growth inhibition of E. coli TolC by carolacton (circles), 20 µg/ml PAβN (triangles), or 40 µg/ml PAβN (squares). (B) Inhibition of E. coli K-12 MG1655 by carolacton (circles), 20 µg/ml PAβN (hexagons), or 40 µg/ml PAβN (diamonds). Carolacton was added in the experiments shown in panels A and B at a final concentration of 0.25 µg/ml where indicated. The figure is representative of the results of three independent biological replicates.

The observed growth inhibition characteristics of carolacton- and PAβN-treated cultures of E. coli TolC and E. coli MG1655 were also reflected in drastic changes in the maximal doubling time (*t*_D_) of cells during exponential growth (Table 3). The *t*_D_ of E. coli TolC after treatment with carolacton increased from 25 to >372 min (Fig. 4 and 7). A comparable decrease of the doubling time was also observed after coadministration of PAβN and carolacton to cultures of E. coli MG1655 (*t*_D_, ~257 min), supporting the previous observation that PAβN treatment can facilitate a carolacton-dependent slowdown of cell division and consequently growth inhibition of an otherwise-resistant strain.

**TABLE 3:** Effect of carolacton treatment and PAβN on growth kinetics of the E. coli strains

E. coli strain and treatment^a^	Maximum specific growth rate (µ_max_, h^−1^)		Doubling time (min)	
	Control	Carolacton^b^	Control	Carolacton^b^
MG1655	1.65 (±0.03)	1.67 (±0.05)	25.1 (±0.4)	25.0 (±0.7)
MG1655, 40 µg/ml PAβN	0.77 (±0.05)	0.17 (±0.05)**	54.3 (±3.6)	257.2 (±59)*
TolC	1.66 (±0.05)	0.11 (±10^−3^)**	25.0 (±0.7)	372.5 (±6)**
TolC, 40 µg/ml PAβN	0.82 (±0.05)	0.05 (±10^−3^)**	51.0 (±2.9)	899.5 (±44)**
TolC, *epi*-carolacton	1.68 (±0.01)	1.67 (±0.02)	24.7 (±0.2)	24.9 (±0.3)
TolC/pIB166-*tolC*	1.63 (±0.19)	1.54 (±0.2)	25.9 (±3.1)	27.4 (±3.5)
TolC/pIB166-*tolC*, 40 µg/ml PAβN	0.75 (±0.04)	0.11 (±10^−3^)**	55.4 (±0.6)	451.9 (±190)

a Carolacton was applied at a final concentration of 0.25 µg/ml, except when *epi*-carolacton was used for treatment at the same final concentration.

b Values in boldface were significantly different from the control, based on a two-tailed Student’s *t* test. **, *P* ≤ 0.001; *, *P* ≤ 0.01.

## DISCUSSION

Here we studied the role of TolC, a component of the major multidrug efflux system of E. coli, in its susceptibility to carolacton. To this end, we determined the genome sequence of the genetically uncharacterized, highly carolacton-susceptible E. coli TolC strain and revealed that it (i) shares the highest nucleotide sequence homology with E. coli MG1655 and (ii) is also phylogenetically reliably placed in a highly supported group that primarily harbors other K-12 strains. Originally, in the 1950s, the chromosome of the wild-type E. coli K-12 was cured from phage λ, generating E. coli K-12 W1485. E. coli K-12 W1485 was subsequently cured of its F^+^ factor to make MG1655 (36). Thus, as E. coli TolC still contains the phage λ and a chromosomal copy of the F plasmid, our TolC strain appears to be an ancient prototrophic derivative of the original wild-type E. coli K-12. The profile of MIC resistance of E. coli TolC provided further evidence for an impairment of the efflux function in the mutant strain, rather than a change in the permeability of the outer membrane (25). As the biological function of the TolC OMP is of great scientific interest, *tolC* deletion mutants of E. coli are often generated anew, elaborately and with varied techniques for every study and in different, often-undescribed genetic backgrounds (25, 27, 28). The E. coli TolC strain sequenced here has now been thoroughly characterized. It is closely related to the ancestral E. coli wild-type strain K-12 and publicly available and thus could be used as a standard tool in the future.

A strong growth inhibition of E. coli TolC occurred at 0.25 µg/ml (0.54 µM). At this concentration, growth of S. pneumoniae TIGR4 is inhibited in a similar way, indicating a bacteriostatic role of carolacton (24). The same concentration of carolacton caused cell death in biofilms of S. mutans (20). A carolacton epimer, C-9 (*R*) (*epi*-carolacton), lacked biological activity in all organisms tested so far (22, 24). Here, we showed that it was also inactive when testing growth of the highly carolacton-sensitive E. coli TolC strain. The complete loss of biological activity of this carolacton derivative, with a mere inversion of a single stereogenic center at C-9, indicates a specific interaction of carolacton with a cellular target. A target that is present not only in streptococci (24) but also in Gram-negative bacteria like *Aggregatibacter* (22) and E. coli, and thus might be conserved in the phyla *Firmicutes* and *Proteobacteria*.

The data demonstrate that carolacton can enter the Gram-negative cell but is a substrate of the tripartite multidrug efflux pump AcrAB-TolC, the main component of intrinsic antibiotic resistance in *Enterobacteriaceae*. Its clinical application would therefore require high concentrations, or could be combined with efflux pump inhibitors. Treatment of the E. coli MG1655 with 40 µg/ml of PAβN, specific for inhibition of the AcrAB-TolC and AcrEF-TolC efflux complexes (16), rendered the strain susceptible to carolacton in a similar way as the deletion of TolC. The effect of AcrEF for the export of carolacton can be neglected here, as its expression is very low and this exporter has a primary role in cell division (37); hence, deletion of *acrEF* does not change the antibiotic resistance phenotype of E. coli (11). Interestingly, lower concentrations of PAβN did not influence the sensitivity to carolacton at all, which is puzzling, because carolacton was provided at 0.25 µg/ml and inhibition by PAβN has been reported to be competitive (16).

The RNA-seq data for E. coli TolC indicated a strong regulatory response upon treatment with carolacton within the first 30 min, where growth is still unaffected, confirming the entry of carolacton into the cell and its likely immediate interaction with an intracellular target. The observed changes involved small regulatory RNAs, a sigma factor, chaperones, heat and cold shock proteins, flagellar components, and membrane transport proteins. The sigma factor F (σ^28^ in E. coli) is needed for flagellar assembly and motility (38), in accordance with the upregulation of the flagellar components *fliL* (*btd92_01821*) or *fliJ* (*btd92_01823*). Interestingly, all ncRNAs of the StyR-44 family were strongly upregulated already at the 5-min time point. Styr-44 ncRNAs are found in ribosomal operons located upstream of the 23S rRNA; their expression is dependent on the growth rate, but their specific function is unknown (39). As ncRNAs are known to act as global regulators of gene expression (40), their differential transcript abundance shows a fast and strong global regulatory response to carolacton. Carolacton treatment also caused upregulation of the outer membrane pore proteins NmpC (log_2_ FC, 2.72) and PhoE (log_2_ FC, 2.85), both of which play a role under heat shock and phosphorus starvation conditions, respectively (41, 42). The transcriptome data showed that the molecular target of carolacton may be located within a central metabolic pathway in the cell, and inhibition of this target induces multiple metabolic and transcriptional adaptations.

In conclusion, we found that carolacton efficiently penetrates the Gram-negative cell envelope, and low micromolar concentrations are sufficient for growth inhibition of E. coli, unless it is exported by the tripartite AcrAB-TolC efflux system. Carolacton might potentially be used against Gram-negative bacteria in combination with EPIs.

## MATERIALS AND METHODS

### Bacterial strains and growth conditions.

E. coli strains used for growth experiments (Table 4) were routinely grown under aerobic conditions in Luria-Bertani (LB) broth overnight (o/n) at 37°C (200 rpm). The cultures were then used to inoculate fresh LB medium to an OD_600_ of 0.05, which was determined photospectrometrically (Ultrospec 3100 Pro; Amersham Biosciences, Inc.). Cultures with an OD_600_ of >0.5 were diluted in LB broth to below 0.5 in order to maintain the linearity between the measured absorbance and cell density and to achieve the most exact results. The initial culture was then split into equal volumes and supplemented with carolacton, 9(*R*) *epi*-carolacton, or PAβN, or maintained as untreated controls. For cryo-conservation, E. coli was grown in LB o/n, mixed with an equal volume of 50% (vol/vol) glycerol in cryovials, and frozen at −80°C.

**TABLE 4:** E. coli strains and plasmids used in this study

Strain or plasmid	Relevant genotype or description	Reference or source
Strains		
DH5α	Cloning strain	Stratagene
K-12 MG1655	F^−^ λ^−^ Δ*ilvG rfb-50 rph-1*	DSM 18039
TolC	F^+^ λ^+^ Δ*ilvG rfb-50 ropS* (33Am) Δ*tolC*	Laboratory collection, DSM 104619
TolC/pIB166-*tolC*	TolC strain containing pIB166-*tolC* for complementation of strain TolC, Cm^r^	This work
Plasmids		
pIB166	Cm^r^	69
pIB166-*tolC*	Removal of P_23_ and integration of *tolC* under control of its native promoter (P_tolC_-*tolC*), Cm^r^	This work

### Storage of carolacton, *epi*-carolacton, and PAβN.

Carolacton and its derivative 9(*R*)-carolacton were dissolved in methanol or DMSO to a final concentration of 5.3 mM (250 µg/ml) or 2 mM (94.3 µg/ml), respectively, and stored in small aliquots in amber glass vials at −20°C in the dark. PAβN (25 mg/ml in H_2_O) was stored at −20°C and used at final concentrations between 5 and 40 µg/ml, as indicated.

### Complementation of E. coli TolC.

Chemo-competent cells of E. coli were prepared according to the TSS method described by Chung et al. (43). pIB166 was PCR amplified with Phusion polymerase (NEB) using primers (pIB166_fwd and pIB166_rev), thereby eliminating P_23_ (Table 5). Genomic DNA of E. coli K-12 MG1655 served as a template for PCR amplification of the *tolC* locus (*b3035*), using primers (tolC_fwd and tolC_rev), additionally introducing flanks homologous to the linearized vector sequence. PCR products were purified with a PCR purification kit (Qiagen, Germany). The PCR-amplified *tolC* gene was cloned into pIB166 by using the CloneEZ kit (Genescript), and the reaction mix was transformed into E. coli DH5α. Obtained plasmids were verified by sequencing and subsequently transformed into E. coli TolC. E. coli transformed with pIB166 or its derivatives were grown on LB agar plates or in liquid LB broth containing 20 µg/ml chloramphenicol.

**TABLE 5:** Overview of oligonucleotides used

Primer	Sequence (5′–3′)	Purpose	Reference
pIB166_fwd	AATTCTAGAGCTCGAGATCTATCGATAAGC	Linearization of pIB166	This work
pIB166_fwd	CAGTCTTAGGTCTGATTTTTTATTTCTATTATTTAC		
tolC_fwd	ATCAGACCTAAGACTGAATGTCCTGGCACTAATAGTGAATTAAATGTGAATTTC	Cloning of *tolC* (*b3035*) of E. coli K-12 MG1655	This work
tolC_rev	CTCGAGCTCTAGAATTTCAGTTACGGAAAGGGTTATGACCGTTACTGGTGGT		

### Determination of MIC values.

MIC values of selected antibiotics and of carolacton against E. coli and E. coli K-12 MG1655 were determined by 2-fold serial microdilution in LB broth with incubation at 37°C for 20 h, as described previously (44). Antibiotics were tested in the following dilution ranges: ampicillin (32 to 0.25 µg/ml), carolacton (8 to 0.03 µg/ml), cephalotin (32 to 0.25 µg/ml), cefotaxime (1 to 0.078 µg/ml), cerulenin (32 to 0.25 µg/ml), ciprofloxacin (0.25 to 0.0019 µg/ml), chloramphenicol (64 to 0.5 µg/ml), corallopyronin A (32 to 0.25 µg/ml), erythromycin (64 to 0.5 µg/ml), gentamicin (32 to 0.25 µg/ml), kanamycin (8 to 0.03 µg/ml), novobiocin (16 to 0.125 µg/ml), penicillin G (32 to 0.25 µg/ml), phosphomycin (32 to 0.25 µg/ml), rifampin (32 to 0.25 µg/ml), sorangicin (32 to 0.25 µg/ml), sulfamethoxazole (256 to 2 µg/ml), triclosan (1 to 0.078 µg/ml), trimethoprim (2 to 0.015 µg/ml), and vancomycin (256 to 2 µg/ml), if not indicated otherwise. Corallopyronin A and sorangicin were kindly provided by Rolf Jansen (HZI, Braunschweig). Antibiotics were purchased from Sigma-Aldrich (Steinheim, Germany) or Carl Roth GmbH (Karlsruhe, Germany). MICs were the lowest concentrations that did not yield visible bacterial growth. The cell count of the initial inoculum was 5 × 10^5^ CFU/ml, which was confirmed by plating of serial cell dilutions and counting of CFU. MICs were confirmed in at least two independent experiments.

### Growth kinetics.

**FIG 8:**
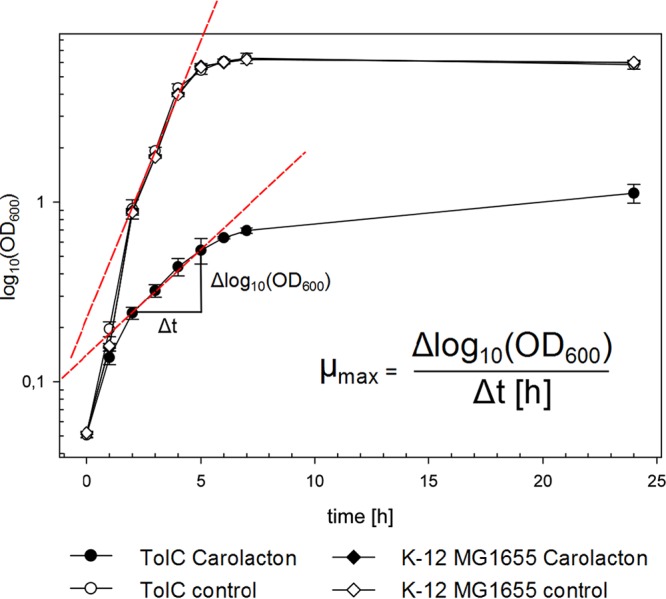
Example of the determination of the maximal specific growth rates of E. coli strains. The maximal specific growth rate (μ_max_, per hour) was calculated from semilogarithmically transformed growth curves according to methods described previously (45).

The maximal specific growth rate (µ_max_, per hour) and doubling time (*t*_D_, in minutes) of bacteria were determined from semilogarithmically transformed growth curves (Fig. 8) according to methods described previously (45).

### Extraction of genomic DNA and PacBio/Illumina sequencing.

Genomic DNA of E. coli TolC was extracted by gravity flow using the Genomic-tip 20/G kit (Qiagen, Germany). Purified genomic DNA of E. coli TolC was processed for PacBio SMRT sequencing and Illumina MiSeq paired-end sequencing (2 × 250 bp) with a target genome coverage of 150-fold. DNA libraries for MiSeq sequencing of the genome of E. coli TolC were prepared with the NEBNext Ultra DNA library prep kit for Illumina sequencing (New England Biolabs, Ipswich, MA). Quality controls of NEBNext Ultra DNA libraries were conducted by fluorometric quantitation using the Qubit 3.0 fluorometer (Thermo, Fisher Scientific, Germany). For PacBio SMRT sequencing, a PacBio SMRTbell library was constructed according to the manufacturer’s instructions and the library was sequenced on the PacBio RSII platform. *De novo* genome assemblies were built with PacBio’s SMRT Portal (v.2.3.0) by utilizing the Hierarchical Genome Assembly Process 3 (HGAP3) (46). The genome was error corrected against indel errors by a mapping of Illumina reads onto finished genomes, using BWA (47) with subsequent variant and consensus calling using VarScan (48); automated sequence annotation was performed with Prokka (v.1.8) (49).

### RNA isolation.

Overnight cultures of E. coli TolC (OD_600_, ~5) were diluted 1:200 in LB broth and grown to an OD_600_ of 0.1. The culture was subsequently divided into equal parts: one part was treated with 0.25 µg/ml carolacton, and the other part was treated with an equal volume of solvent (methanol). Cells were sampled before treatment and at 5, 15, and 30 min post-addition of carolacton. The samples were transferred to an equal volume of RNAProtect (Qiagen, Germany) and incubated for 5 min at room temperature. Cells were pelleted (13,000 rpm, 2 min), the supernatant was removed, and the pellet was frozen at −80°C. For RNA extraction, the pellets were washed with 0.5 ml nuclease-free water and centrifuged (13,000 rpm, 2 min). RNA extraction was carried out using the miRNeasy minikit (Qiagen, Germany) according to the manufacturer’s instructions for purification of total RNA. The removal of genomic DNA was carried out by the optional on-column DNase I digestion using the DNase I kit (Qiagen, Germany) for 45 min. After the washing steps, the RNA was eluted in 50 µl of nuclease-free water supplied with the kit. To test the integrity of the isolated total RNA and the enriched mRNA, samples were analyzed using the Agilent 2100 Bioanalyzer and the RNA 6000 Pico kit (Agilent, Germany).

### Enrichment of mRNA and RNA sequencing.

mRNA enrichment was achieved by using the RiboZero kit for Gram-negative bacteria (epicenter; Illumina) for 2 µg of total RNA as described by the manufacturer. Successful removal of rRNA was verified using an Agilent 2100 Bioanalyzer (Agilent, Germany). Direct strand-specific RNA sequencing was performed using the Illumina HiSeq 2500 platform (Illumina) according to the ScriptSeq v.2 protocol for RNA-seq library construction (Agilent, Germany). After quality control and clipping of adapter sequences (primers and bar codes), mapping of reads and data analysis was conducted using the Rockhopper software (v.2.0.3) (50).

### RNA-seq data analysis.

Trimming of Illumina sequencing adapter sequences of obtained reads was achieved using fastq-mcf (51). Reads were mapped to the E. coli TolC genome (CP018801.1), and the read counts per feature were determined with Rockhopper (v.2.0.3) (50, 52). For analysis of differential abundance of transcripts, the raw read counts obtained with Rockhopper (53) were used, and changes in transcript abundance levels were calculated with the Bioconductor edgeR package (v.3.1) for R (v.3.10.0) (54, 55). False-discovery rate (FDR)-adjusted *P* values were calculated according to methods described previously (56). FDR values of <0.01 were considered significant. Heat maps were generated for genes that showed a log_2_ FC of ≥±2 for at least one time point (FDR, ≤0.01), log_2_ FC values of transcript abundance obtained with edgeR were used as input for the heatmap.2 function of the R package gplots (v.2.15.0) (57).

### Whole-genome-based phylogenomic analyses.

To elucidate the phylogenetic positioning of strain TolC, and given its high sequence similarity to strain E. coli K-12 MG1655, a member of phylogroup A (58), a corresponding reference data set was defined. The latter included all 32 members of phylogroup A, according to methods described previously (58), and was further complemented by four recently genome-sequenced strains that had been found to be highly similar to TolC (accession numbers NZ_CP011495, NZ_CP014225, NZ_CP014348, and NZ_CP016018). Two whole-genome-based phylogenomic analyses were conducted using the genome BLAST distance phylogeny approach (59) in its latest version (29). The first analysis was based on the nucleotide data restricted to genes, whereas the second one used protein data only. Coding regions were determined via Prodigal under default settings (60). All pairwise intergenomic distances were calculated with GBDP under established settings (58), i.e., using the trimming algorithm, distance formula d_5_, and an E value cutoff of 10^−8^. A total of 100 pseudobootstrap replicates were calculated per distance and later used for the inference of branch support values (61). Phylogenetic trees were inferred from the original and pseudobootstrapped distance matrices by using FastME 2.1.4 (62) under the SPR branch-swapping option and rooted using the midpoint method (63).

Software GBDP-based *in silico* DNA-DNA hybridization was achieved with the online version of the Genome-to-Genome Distance Calculator (GGDC v.2.1; http://ggdc.dsmz.de) (29), using the output of formula 2 (i.e., robust against the use of incomplete genome sequences), as recommended by the software creators. Whole-genome comparisons between E. coli strains were conducted with the BLAST Ring Image Generator (v.0.95) (64) and Easyfig (v.2.2.2) (65), both of which utilize BLAST+ (v.2.5.0) (66).

### Accession number(s).

The genome sequences of E. coli TolC were deposited in NCBI’s GenBank (67) under accession number CP018801.1. Raw and processed RNA-seq data were deposited in NCBI’s Gene Expression Omnibus (GEO) database (68) and are accessible through GEO Series accession number GSE93125.
